# Study of Bone Marrow Mesenchymal and Tendon-Derived Stem Cells Transplantation on the Regenerating Effect of Achilles Tendon Ruptures in Rats

**DOI:** 10.1155/2015/984146

**Published:** 2015-08-03

**Authors:** Mohanad Kh Al-ani, Kang Xu, Yanjun Sun, Lianhong Pan, ZhiLing Xu, Li Yang

**Affiliations:** ^1^Key Laboratory of Biorheological Science and Technology, Ministry of Education, Bioengineering College, Chongqing University, Chongqing 400030, China; ^2^Veterinary College, Tikrit University, Ministry of Higher Education, Tikrit, Iraq

## Abstract

Comparative therapeutic significance of tendon-derived stem cells (TDSCs) and bone marrow mesenchymal stem cells (BMSCs) transplantation to treat ruptured Achilles tendon was studied. Three groups of SD rats comprising 24 rats each, designated as TDSCs and BMSCs, and nontreated were studied for regenerative effects through morpho-histological evaluations and ultimate failure load. For possible mechanism in tendon repair/regeneration through TDSCs and BMSCs, we measured Collagen-I (Col-I), Col-III gene expression level by RT-PCR, and Tenascin-C expression via immunofluorescent assay. TDSCs showed higher agility in tendon healing with better appearance density and well-organized longitudinal fibrous structure, though BMSCs also showed positive effects. Initially the ultimate failure load was considerably higher in TDSCs than other two study groups during the weeks 1 and 2, but at week 4 it attained an average or healthy tendon strength of 30.2 N. Similar higher tendency in Col-I/III gene expression level during weeks 1, 2, and 4 was observed in TDSCs treated group with an upregulation of 1.5-fold and 1.1-fold than the other two study groups. Immunofluorescent assay revealed higher expression of Tenascin-C in TDSCs at week 1, while both TDSCs and BMSCs treated groups showed detectable CM-Dil-labelled cells at week 4. Compared with BMSCs, TDSCs showed higher regenerative potential while treating ruptured Achilles tendons in rats.

## 1. Introduction

About 30 million ligament and tendon injuries are reported annually across the globe due to lifestyle, recreation, work patterns, accidents, pharmacological agents, and degenerative biological variables such as gender, age, and genetics [1]. Anatomical studies are crucial for in-depth understanding of tendon healing and regeneration. Tendon is comprised of parallel and well organized collagen (Col) bundles, of which approximately 90% are of Col-I, while the rest of the 10% are Col-III, -IV, -V, and -VI [2]. Chronic or acute tendon injuries are primarily treated with conservative or surgical treatments, where the former is used for symptomatic relief only, is ineffective and time-consuming, but later involves the use of autografts, allografts, xenografts, and prosthetic devices [3, 4]. However, there are considerably high risks of complications such as infection, nerve damage, adhesion, and distributed skin sensibility. Therefore, it is crucial to define some innovative techniques to treat such tendon injuries.

Stem cells are undifferentiated and self-renewing cells able to differentiate into specialized cells of different types with specific functions including the biological healing process [5, 6] and include TDSCs, BMSCs, adipose-derived mesenchymal stem cells (AdMSCs), and umbilical cord blood-derived stem cells (UCB-SCs). TDSCs and BMSCs have various advantages and superiority over the other many different stem cell types such as quick proliferation, tendon repair specificity, reduced regeneration time, and superior shaped tendon formation [7]; hence we used these two stem cell types as a model in the present study. Recently various researchers reported that BMSCs may differentiate to give rise into several connective tissue types including bone, cartilage, tendon, muscle, marrow, fat, and dermis [8–10]. BMSCs are involved to facilitate the tendon healing process intrinsically by accelerating fibroblast proliferation and modulation of certain growth factors and cytokinins [11–13]. On the other hand, TDSCs are adult stem cells residing in tendons [14], which are histologically and biochemically proven as a prime source of tendon repair [15–17]. Cheng and coworkers demonstrated that TDSCs have high potential for colony-formation compared with BMSCs [18]. It is also said that TDSCs expresses higher mRNA level of tenogenic markers- scleraxis (*Scx*), tenomodulin (*Tnmd*), and extracellular matrix (ECM) components of tendon, that is, Col-1A1, Col-1A1/Cl3-A1 ratio, and decorin (*Dcn*), than BMSCs. However, a comparison between TDSCs and BMSCs on treating tendon injury has not yet been done. We hypothesize that TDSCs are more favorable for treating Achilles tendon injuries.

In the present study, we transplanted TDSCs and BMSCs into the ruptured area of the Achilles tendon for macroscopic appearance; histomorphological analyses and biomechanical strength were observed to find the possible mechanism of the repair promotion and evaluated the cell transplantation effects of both stem cell types. The results indicate that TDSCs exhibit a better-regenerative potential when compared with BMSCs in treating ruptured Achilles tendons and could be a better alternative cell source for treating Achilles tendon.

## 2. Materials and Methods

### 2.1. Ethics Statement

Ethics Committee of Chongqing University, College of Bioengineering, and Daping Hospital Animal Experimental Center approved all experimental protocols using SD rats including collection of Achilles tendon samples.

### 2.2. Rats and Treatment Groups

Seventy-eight Sprague-Dawley (SD) male rats weighing 200 g obtained from the Daping Hospital's animal experimental center (Chongqing, China) were used as recipients or donors. Six SD rats, which did not undergo an operation, were the source of tendon-derived stem cells, bone marrow mesenchymal stem cells, and healthy Achilles tendons. The remaining 72 SD rats were used for Achilles tendon healing experiments. The rats were divided in to three groups: TDSC, BMSC, and nontreated group, each group 24 rats. The study was carried out at three time points 1 week, 2 weeks, and 4 weeks. Eight rats were assigned to each time point. The rats were placed in individual cages under slandered feeding system.

### 2.3. Cell Isolation

Six SD rats were used to isolate the various cells. The rats were sedated with pentobarbital sodium in an anesthetic chamber. Then, using 3% Fluothane in a mask, they were sacrificed. The femur and tibia, including the tendons attached to them, were dissected. BMSCs of SD rats were isolated using a modified procedure [18]. Briefly, both femur and tibia were excised, and the diaphyses were cut. The bone marrow was flushed out with DMEM-LG (Gibco) supplemented with 10% FBS, 100 U/mL penicillin, and 100 *μ*g/mL streptomycin. Single cell suspension was generated by aspirating the bone marrow back through the syringe. The samples were then washed and centrifuged at 1000 rpm to remove the pieces of debris. The cell pellets were resuspended and expanded in a humidified incubator at 5% CO_2_ and 37°C. TDSCs were isolated from rats by removal of the tendons and rinsed with PBS. The TDSCs were isolated according to the previous report [19]. Briefly, the Achilles tendons were then minced into small pieces and digested with 5 mg/mL of type I collagenase (SIGMA) at 37°C with 5% CO_2_ for two hours. The undigested tissues were removed by using 70 mm nylon sieve, and the remaining cell pellets were cultured with low glucose Dulbecco's modified Eagle's medium (DMEM, Gibco) L-glutamine, 10% FBS, 100 U/mL penicillin, and 100 *μ*g/mL streptomycin. After 12 days, the cell colonies formed and were selected for further culture. After 100% confluence, cells were subcultured, and the medium was changed every third day. For both BMSCs and TDSCs, Passage 3 (P3) cells were adopted for identification.

### 2.4. Cell Identification and Multidifferentiation Assays

Flow cytometry was used for identifying stem cell surface markers: CD29, CD44, and CD90 of TDSCs and BMSCs. The osteogenic, adipogenic, and chondrogenic differentiation potential of TDSCs and BMSCs were tested according to the previous report [19, 20]. After induction, Alizarin red, Oil red, and Toluidine blue staining assays were used to confirm osteogenesis, adipogenesis, and chondrogenesis, respectively.

### 2.5. Animal Model and Surgical Procedures

**Figure 1:**
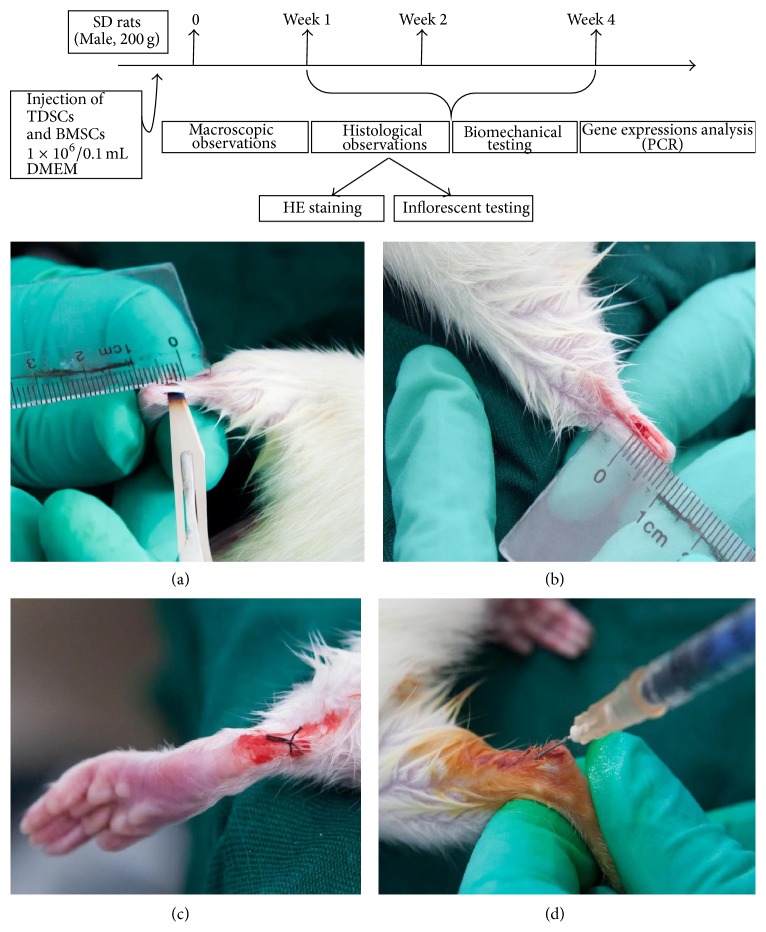
Experimental protocol. A complete transverse incision was made 10 mm from the calcaneal insertion of the Achilles tendon ((a) and (b)). Clinical suture was used to suture the skin and iodine was directly applied (c). TDSCs (1 × 10^6^/0.1 mL DMEM) or BMSCs (1 × 10^6^/0.1 mL DMEM) were injected in the Achilles tendon with use of a syringe (d).

Seventy-two SD rats, weighing 200 g, provided by Daping Hospital and the Research Institute of Surgery of the Third Military Medical University were used. Prior to the study, all operations and handling procedures were approved by the hospital. The rats were divided into three groups: the BMSCs group, the TDSCs group, and the nontreated group, each group 24 rats. Three rats from each group were tested in each time point. The left hind legs of the animals were used for micro/macro observation while the right hind legs were used for measuring gene expression. Five rats from each group from each time point were used for biomechanical evaluation. The rats were anesthetized with pentobarbital sodium in an anesthetic chamber and then with 3% Fluothane in a mask. In aseptic conditions, 10 mm longitudinal incision of the right and left hind limb was made directly over the Achilles tendon (Figures 1(a) and 1(b)). A segment from the middle part of the Achilles tendon was cut using a surgical blade 5 mm from the calcaneal insertion site. Clinical sutures were used to suture the incision, and iodine was directly applied (Figure 1(c)). The TDSCs and BMSCs were labeled with CM-DiI (C7000, Invitrogen), after that the donor TDSCs (1 × 10^6^/0.1 mL DMEM) or BMSCs (1 × 10^6^/0.1 mL DMEM) were injected around the Achilles tendon of each rat with a syringe (Figure 1(d)). During the recovery period, the rats were placed in individually sterilized cages under standard feeding system. At the end of each time point, the rats were sacrificed by over dose of ether anesthesia.

### 2.6. Macroscopic Assessment

At the end of each time point, the rats were sacrificed, and the treated legs were removed for macroscopic observation. The appearance of the regenerated tendons was observed and compared to that of the nontreated group.

### 2.7. Histological Evaluation

The treated rats were sacrificed, and the Achilles tendon between the calcaneus and musculotendinous junction was harvested at each time point. The tendon was immersed in 4% PFA overnight, dehydrated, and embedded into optimal cutting temperature compound (OCT). The specimens were cut into 10 *μ*m sections by freezing microtome (Leica CM1900) and stained with hematoxylin and eosin (HE) for histological evaluation.

### 2.8. Biomechanical Testing

Five rats from each group were used for biomechanical testing as follows: the Achilles tendon between the calcaneus and musculotendinous junction resected at 1 week, 2 weeks, and 4 weeks after incision. The proximal and distal ends of the Achilles tendon were fixed securely in serrated grips and mounted on to a mechanical testing machine (Instron). With 100 N load cell capacity, the Achilles tendon was pulled at a constant speed of 10 mm/min until rupture. The data was recorded with software (Win Test'7).

### 2.9. Quantitative (RT) Polymerase Chain Reaction (qPCR)

We examined the expression of Col-III, Col-I, and GAPDH in every time point. Total RNA was extracted using the total RNA extraction kit (Bioteke Corporation) according to the manufacturer's instructions. RNA was subjected to reverse transcription to complementary DNA (cDNA) using the First Strand cDNA kit (Thermo Scientific Rt-First Strand cDNA Synthesis kit, K1622). PCR conditions were 65°C for 5 min, then 42°C for 60 min, and termination at 70°C for 5 min. The products were stored at 80°C. Total cDNA for each sample was amplified in a final volume of the reaction mixture containing SsoAdvanced SYBR Green qRT-PCR supermix (Bio-Rad number 1725264) ready-to-use reaction cocktail and specific primers for Col-I: Forward: AAGGTGACAGAGGCATAAAG, Reverse: GGAAGCTGAAGTCATAACCA And Col-III: Forward: CATGATGAGCTTTGTGCAAT, Reverse: CTGCTGTGCCAAAATAAGAG. The cycling conditions were the denaturation at 95°C for 30 sec, 39 cycles at 95°C for 5 sec, optimal annealing temperature for 20 sec, 72°C for 30 sec, and 60°C to 95°C with a heating rate of 0.1°C/s. The CFX 96 Real-Time PCR Detection System (Bio-Rad) was used to record the results. The relative expression level of the gene of interest normalized to GAPDH was analyzed according to the 2^−ΔΔct^ Method.

### 2.10. Immunofluorescent Assay

The treated Achilles tendon of each group was collected in week 1 and week 4 and performed frozen sections. All the sections were immune stained with Tenascin-C (1 : 100, Abcam) primary antibody, followed by Alexa Fluor 488 dye-labeled secondary antibody. DAPI (Roche) was used to stain cell nuclei and observed under the immunofluorescent microscope to check the cells transplantation regenerative processes.

### 2.11. Statistical Analysis

All the data are expressed as means ± standard deviations. Statistical analysis was performed with one-way ANOVA, followed by LSD test for comparison between two groups (Origin Lab Origin V 8.0 Software). A *P* value of <0.05 was considered significant.

## 3. Results

### 3.1. Study of Stem Cell Markers for the Confirmation and Differentiation Ability of TDSCs and BMSCs

**Figure 2:**
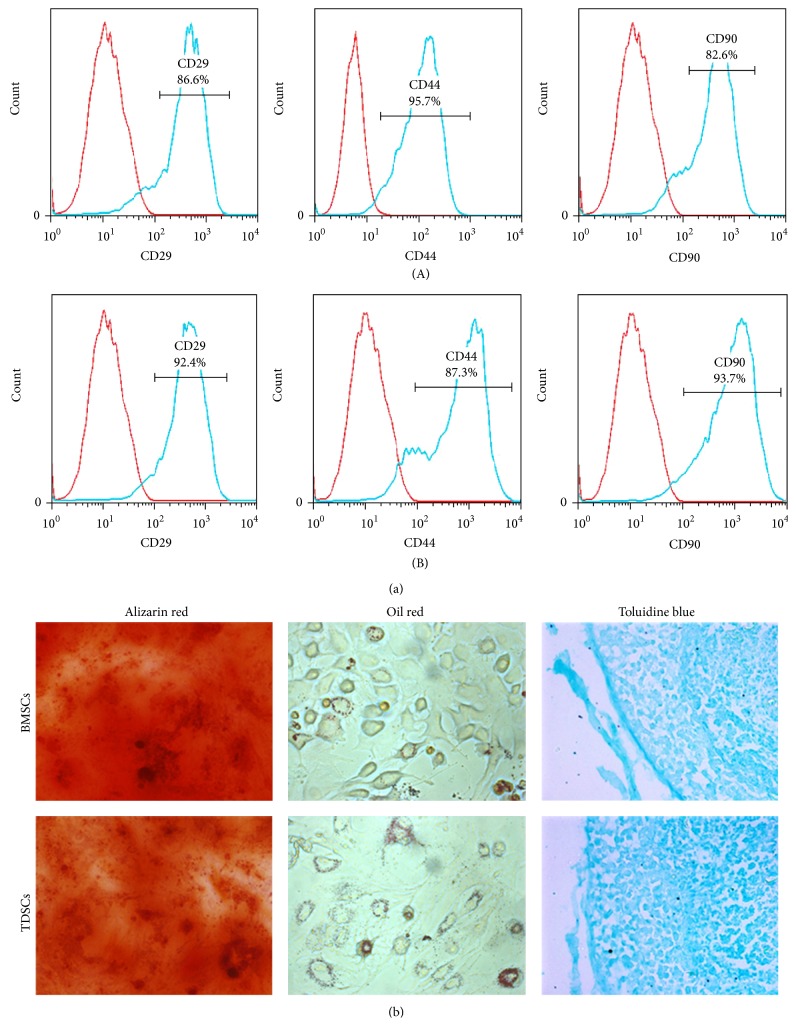
Cell identification of TDSCs and BMSCs. (a) Stem cell surface markers of FCM: (a)-(A) TDSCs, (a)-(B) BMSCs. (b) Multidifferentiation of TDSCs and BMSCs: osteogenesis (Alizarin red); adipogenesis (Oil red); chondrogenic (Toluidine blue).

Flow cytometry analysis was done to identify the stem cells. Both TDSCs and BMSCs were tested positively for CD 29, CD 44, and CD 90, which are indicative of stem cell surface markers (Figure 2(a)). It confirmed that the cells we isolated from the Achilles tendon and bone marrow were stem cells. Multidifferentiation capacity is one of the universal characteristics of stem cells. The differentiation analyses showed that the adipogenesis (Oil red), chondrogenesis (Toluidine blue), and osteogenesis (Alizarin red) of the isolated TDSCs and BMSCs were emerged after induction (Figure 2(b)). Hence, based on the results of cell identification analyses, it became assured that the isolated stem cells were TDSCs and BMSCs.

### 3.2. Week-Wise Macroscopic Assessment of Morphological Changes in Repaired Achilles Tendon

**Figure 3:**
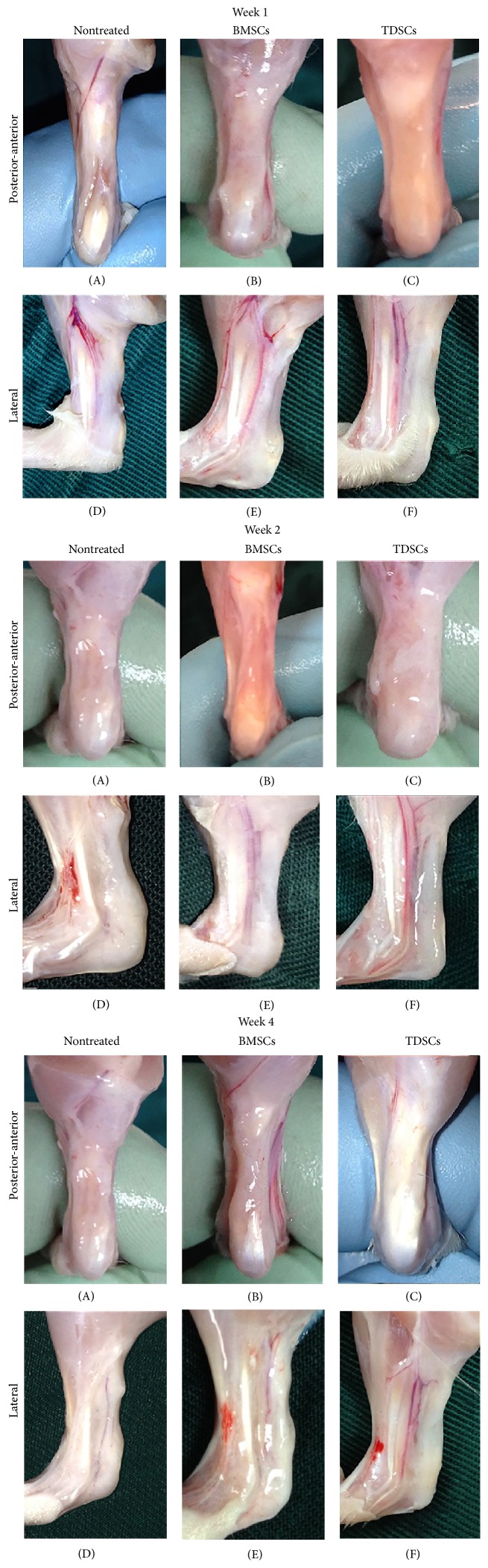
Macroscopic findings at 1 w, 2 w, and 4 w after surgery. The posterior-anterior appearance of the Achilles tendon in the nontreated group (A), the BMSCs group (B), and the TDSCs group (C). The lateral appearance of the Achilles tendon in the nontreated group (D), BMSCs group (E), and TDSCs group (F).

The skin was sutured with a clinical suture to inject TDSCs (1 × 10^6^/0.1 mL DMEM) and/or BMSCs (1 × 10^6^/0.1 mL DMEM) in the Achilles tendon using a sterile syringe. After transplantation of the TDSCs and BMSCs, changes in appearance on the treated area were analyzed at weeks 1, 2, and 4 after surgeries. At week 1, the defect area appeared clearly in the nontreated group (Figures 3(A) and 3(D)), while the BMSCs group revealed some connective tissue in the treated area (Figures 3(B) and 3(E)). The TDSCs group revealed a good start of growth in connective tissue around the treated area (Figures 3(C) and 3(F)). On approaching week 2, the growth of connective tissue was stunted while the defected place area was obvious in the nontreated group, which remained as such during week 1 (Figures 3(A) and 3(D)). On the other hand, BMSCs treated group appeared better than week 1 group, and TDSCs showed the best growth in connective tissue at the treated area (Figures 3(C) and 3(F)).

At week 4, the TDSCs group appeared significantly different than the other groups and displayed a complete Achilles tendon with a normal appearance in the posterior-anterior and lateral views (Figures 3(C) and 3(F)). The BMSCs group showed obvious connective tissue growth with a little transverse notch appearance clearly in the posterior-anterior and lateral views (Figures 3(B) and 3(E)). The nontreated group showed no tissue growth at all (Figures 3(A) and 3(D)).

### 3.3. Biomechanical Testing

**Figure 4:**
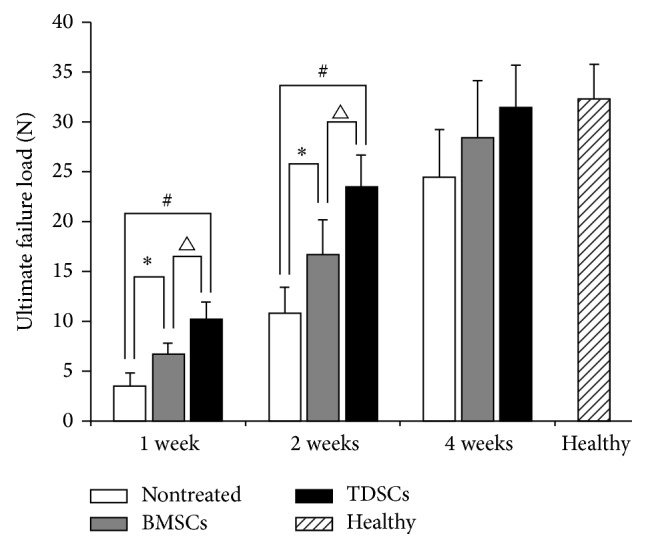
Results of biomechanical testing. The ultimate failure load in the nontreated group, the BMSCs group, and the TDSCs group at 1 w, 2 w, and 4 w after surgery. Healthy indicates the ultimate failure load in a normal Achilles tendon at thirteen weeks of age. ^*^ *P* < 0.05 and ^#^ *P* < 0.05 compared with control and ^▵^ *P* < 0.05 compared with BMSCs were considered significant (data represents mean ± SD, *n* = 5).

The tensile strength of the repaired Achilles tendon in various study groups was tested by the ultimate failure load method. The ultimate failure load in the TDSCs group was considerably higher (10.2 N) than that in BMSCs (6.7 N) and nontreated groups (3.5 N) at week 1 after incision (*P* < 0.05). The failure load of TDSCs and BMSCs was increased 1.9-fold (^#^ *P* < 0.05) and 0.9-fold (^*^ *P* < 0.05), respectively, where the former has 0.5-fold higher change (^▵^ *P* < 0.05). The ultimate failure load at week 2 after incision was significantly higher for the TDSCs (23.5 N) than the BMSCs (16.5 N) and nontreated (10.8 N) by 1.2-fold (^#^ *P* < 0.05) for TDSCs and the BMSCs group by 0.5-fold (^*^ *P* < 0.05). The failure load of TDSCs exceeded 0.5-fold (^▵^ *P* < 0.05) versus BMSCs. At week 4, the ultimate failure load of the TDSCs group again showed a higher value (30.2 N) than the BMSCs group (28.45 N) and the nontreated group (25.3 N). It is important to note that among these three groups, BMSCs were also higher than the nontreated group. However, there was no statistically obvious difference between TDSCs and BMSCs. In addition, at week 4 after surgery, the ultimate failure load of TDSCs and BMSCs reached nearly that of a healthy (Figure 4).

### 3.4. Histological Study of the Healing Achilles Tendon

**Figure 5:**
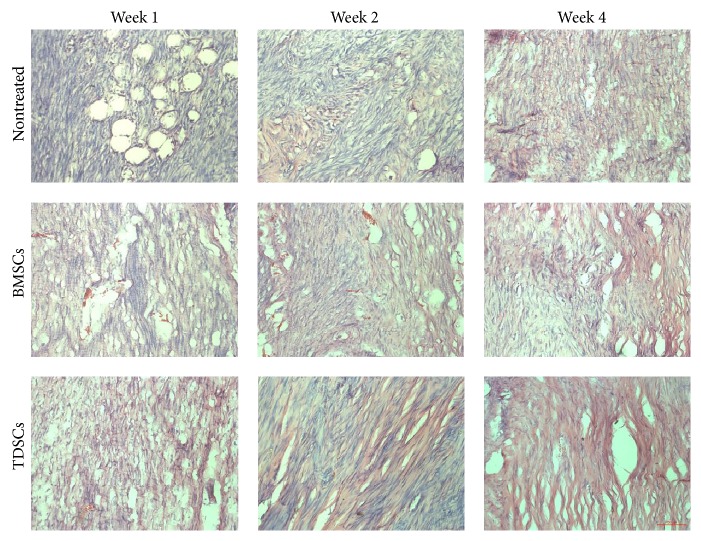
Histological analysis of the Achilles tendon: HE staining of the nontreated group, the BMSCs group, and the TDSCs group, at three time points (1 week, 2 weeks, 4 weeks). Bar: 200 *μ*m.

**Figure 6:**
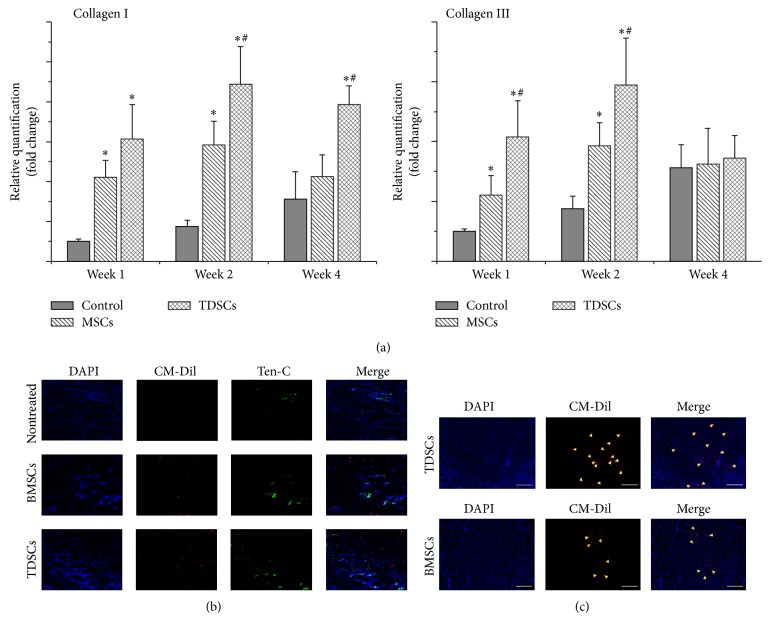
The analysis of tendon-related ECM expression. (a) Rat-specific gene expression analysis of tendon-related ECM genes: collagen I and collagen III. The gene transcript levels were relative to GAPDH and normalized to nontreated group. ^*^ *P* < 0.05 means compared with the control group, ^#^ *P* < 0.05 means compared with BMSCs group were considered significant (data represents mean ± SD, *n* = 3). (b) Tenascin-C immunofluorescent testing in the injured Achilles tendon. (c) Cell tracking of TDSCs and BMSCs after transplant at 4 weeks: the nuclei were stained by DAPI (blue spots); the CM-Dil was red spots. Bar: 200 *μ*m.

The histological analyses of Achilles tendon sections were made and gone through hematoxylin and eosin staining for the nontreated group, the BMSCs group, and the TDSCs group, at three time points (weeks 1, 2, and 4) (Figure 5). Dense connective tissue was observed in the TDSCs and BMSCs treated groups at week 1 after surgery. For the TDSCs group, visible longitudinal fibrous tissue had already emerged along with well-organized cell structures not seen in other two groups. At week 2 after surgery, both TDSCs- and BMSCs-treated and particularly TDSCs-treated tendons exhibited more ECM deposition and obvious longitudinal fibrous tissue than that of nontreated tendons with a greater number of spindle-shaped cells aligned/organized along the longitudinal (tensile) axis of the tendon. A similar trend was observed at week 4 after surgery TDSCs given improved tendon status than BSMCs, and hence both TDSCs and BSMCs showed a spindle-shaped morphology distributed along the longitudinal fibrous tissue of the tendon. On the contrary, the cells of the loose and thin longitudinal fibrous tissue that began to appear in the nontreated group made little organization with higher vascularization. In order to examine the transplanted, labeled TDSCs in the excised Achilles tendon, frozen sections were prepared and analyzed by fluorescent microscopy. The CM-DiI positive cells (red) were detectable around the tendon at week 4 after transplant (Figures 6(a) and 6(b)), indicating that those transplanted cells were still alive and may participate in the process of regeneration.

### 3.5. Collagen I and Collagen III Gene Expression Analysis

To detect host ECM (collagen) deposition conditions, quantitative real-time PCR was performed to investigate rat-specific Col-I and Col-III (Figure 6(a)). At the first week, TDSCs and BMSCs Col-I gene expression levels were upregulated up to 6.2-fold (*P* < 0.05) and 4.2-fold (*P* < 0.05), respectively. The levels of Col-III were upregulated by 4.2-fold (*P* < 0.05) in TDSCs and 2.2-fold (*P* < 0.05) in BMSCs compared with nontreated group. In addition, the expression of Col-I and -III was higher in the TDSCs group than the BMSCs group and it showed significant differences (*P* < 0.05). At week 2 after surgery, Col-I gene expression levels were upregulated by 4.1-fold (*P* < 0.05) and 2.3-fold (*P* < 0.05) in the TDSCs and BMSCs groups, respectively. In addition, in both TDSCs and BSMCs the Col-III levels were upregulated by 2.3-fold (*P* < 0.05) and 1.2-fold (*P* < 0.05), respectively, compared with nontreated group. In addition, the expression of Col-I and -III was higher in the TDSCs group compared with the BMSCs group and it showed significant differences (*P* < 0.05). At week 4 after surgery time, TDSCs Col-I level was higher than BMSCs; it showed a significant difference, but only the TDSCs Col-I gene expression level showed a significant difference and was upregulated by 1.5-fold (*P* < 0.05) compared with nontreated group, and by 1.1-fold (*P* < 0.05) in the BMSCs-treated group (Figure 6(a)). Additionally, Col-III in all groups showed almost the same expression level (Figure 6(a)). It is obvious that both TDSCs and BMSCs have the ability to boost the ECM gene expression. But herein we observed that TDSCs compared to BMSCs have more agility to trigger the genes involved in expression of ECM and accelerated tendon repair many folds.

### 3.6. Immunofluorescent Assay of Injured Achilles Tendon followed by TDSCs and BMSCs Transplantation

The organization by immunofluorescence staining found that, after 1 week of implantation TDSCs and BMSCs in the injured Achilles tendon, cells were found in many parts of the distribution of CM-Dil labelled (Figure 6(b)). Following implantation of TDSCs and BMSCs around the injured Achilles tendon, a large portion with Tenascin-C staining was detected in both treated groups (Figure 6(b)), where TDSCs treated group of mouse showed higher expression of Tenascin-C than the BMSCs group. In addition, 4 weeks after implantation TDSCs and BMSCs were still able to detect CM-Dil labelled cells (Figure 6(c)). In short, the results suggest that in the early postimplantation TDSCs and BMSCs can promote the expression of Tenascin-C. Additionally, the implanted cells can survive for at least 1 month in the Achilles tendon injury and are involved in the tendon reconstruction.

## 4. Discussion

Achilles tendon rupture accounts for about 35% of all tendon injuries due to low blood supply and low metabolic activity of tendon fibroblastic cells, in addition to these low healing potentials seen in the ruptured tendons. Previous studies reported TDSCs to be immune-privileged cells having potential for allogeneic transplantation [2, 14, 16], which led to cell banking and can be used during emergencies. Tissue regeneration depends primarily on the rate of proliferation and differentiation of endogenous stem cells to produce a high amount of ECM. It has been reported that TDSCs have higher colony-formation ability, proliferate rapidly, and possess some universal stem cell characteristic compared to BMSCs depending on the age and origin of the stem cells [17, 19–21]. Studies also show that TDSCs express higher mRNA level of tenogenic markers, scleraxis, tenomodulin, and ECM components of tendon compared to BMSCs [14, 17, 22]. By using mouse model Bi et al. [14] found that, compared to BMSCs, TDSCs have more ability to express higher mRNA level for Sox9, Comp, Runx2, and Scx, while in humans TDSCs express increased level of tenomodulin (TNMD) compared to human BMSCs does.

TDSCs as compared to BMSCs are thought to be a potent therapeutic cell treasure, which take an active part in proper and enhanced musculoskeletal repair including tendon repair [21, 23]. TDSCs showed high potential for chondrogenic and osteogenic differentiation compared to BMSCs and hence at appealing candidate for tendon-bone junction regeneration [21]. Keeping in view the superiority of DMSCs over other stem cell lines, in our study, we chose two different stem cells, namely, TDSCs and BMSCs, and transplanted them into the Achilles tendon injured area of rats. After transplantation, the animals were allowed to heal for four weeks. Macroscopic appearance, histomorphology, and biomechanical strength were used to evaluate animal performance and tissue integrity throughout the healing process. At four weeks into the experiment, the treated (TDSCs and BMSCs) groups showed better results than the nontreated group. In the early stages of regeneration, TDSCs showed a prompt stimulatory effect on tissue remodeling, in both macro/micro appearance and biomechanical strength. At one week after transplantation small changes were observed; regenerated tissue was covering the treated region in the TDSCs and BMSCs groups while there was no visible connective tissue in the injured area of the nontreated group. At two weeks, the TDSCs were better in macro/micro appearance than the other two groups. Four weeks after transplantation, the histological evaluation of TDSCs detected more fibroblastic cell presence arranged in parallel rows and positive collagen fiber in the treated area as seen in the TDSCs group (Figure 5), and the Achilles tendon displayed almost normal appearance (Figures 3(C) and 3(F)).

The higher mechanical strength can be explained by the increasing production of collagen. Higher mechanical strength suggests that the cell transplantation promotes the organization and synthesis of the various ECM components responsible for the structural and functional repair of tendon tissue during different stages of healing. The ultimate failure load in the TDSCs group was considerably higher than that of the BMSCs group and the nontreated group. At one week and two weeks, TDSCs rapidly improved biomechanical strength in the early stages of healing. However, after four weeks, the ultimate failure load of the TDSCs group still showed better results than other groups but was not significantly different. In addition, the TDSCs and BMSCs groups showed a higher value of reached almost the healthy (Figure 4).

In short, we supposed that, after cell transplantation, TDSCs are the first to adapt to the microenvironment in the ruptured Achilles tendon. Because of the fast proliferation and high affinity of tendon niches, TDSCs might differentiate rapidly into the functional tenocytes to synthesize a greater amount of ECM for remodeling.

In addition, we investigate the possible mechanism of the repair promotion by cell transplantation and test it by three experiments, RT-PCR to check the collagen expression, immunofluorescence to analyze the Tenascin-C protein expression, and CM-Dil to locate the stem cell. Initially the gene expression of both Col-I and Col-III genes were upregulated after transplantation of TDSCs and BMSCs in Achilles tendon, and TDSCs showed a higher enhancing effect than BMSCs. We considered that, in the short time since the injury, the host needs a lot of cells to concentrate into the wound. After cell transplantation, TDSCs and BMSCs quickly joined in the regenerative process and revealed a high gene expression level of collagen. However, at 4 weeks, the stimulatory effect of BMSCs for collagen type I gene expression was gone. In contrast, TDSCs still showed the enhanced effect, which means TDSCs provide continuous stimulation of collagen type I for connective tissue formation in the injured area. In addition, at week 4, all the enhancement of Col-III gene expression in the TDSCs and BMSCs was gone. Because of the complicated signal mechanism of the microenvironment after four weeks, Col-III synthesis might not play the most important role in the regenerative process. The explanation may be that Col-III is more important during the earliest stages of tendon healing, because it can rapidly form crosslinks and stabilize the precarious repair site but less so in the late stages of tissue remodeling [15, 22].

Tenascin-C is reported as an important ECM protein in providing elasticity to the musculoskeletal tissues [15, 24]. This feature is of great importance in the degenerative and regenerative processes where the normal biomechanical environment of the musculoskeletal tissues is disturbed by injury. In our study, we found that, in the early stage, both TDSCs and BMSCs implantation groups can promote Tenascin-C protein synthesis in the Achilles tendon ruptured location, and even former was observed with high protein expression than the latter group.

The present study led us to speculate that stem cells transplantation promotes regenerative processes, and TDSCs are the first to adapt within the microenvironment in ruptured Achilles tendon. In addition, we found the CM-Dil labeled transplanted cells were detectable around the tendon at week 4 after surgery. It can be interpreted that the transplanted cells were still alive and were involved in the tendon remodeling process quickly and efficiently as compared to BMSCs and nontreated group, hence proven to be the best choice.

## 5. Conclusion

This study provides evidence that TDSC and BMSC transplantation improves the healing potential of ruptured Achilles tendon in rats. In addition, TDSCs exhibited a better regenerative potential when compared with BMSCs in treating ruptured Achilles tendons and may be a better alternative cell source for treatment during Achilles tendon injuries.
